# Management of *Helicobacter pylori* treatment failures: A large population-based study (HP treatment failures trial)

**DOI:** 10.1371/journal.pone.0294403

**Published:** 2023-11-30

**Authors:** Natsuda Aumpan, Navapan Issariyakulkarn, Varocha Mahachai, David Graham, Yoshio Yamaoka, Ratha-korn Vilaichone

**Affiliations:** 1 Center of Excellence in Digestive Diseases and Gastroenterology Unit, Department of Medicine, Thammasat University, Pathumthani, Thailand; 2 Department of Medicine, Chulabhorn International College of Medicine (CICM) at Thammasat University, Pathumthani, Thailand; 3 Department of Medicine, Michael E. DeBakey VA Medical Center and Baylor College of Medicine, Houston, Texas, United States of America; 4 Department of Environmental and Preventive Medicine, Oita University Faculty of Medicine, Yufu, Japan; 5 Research Center for Global and Local Infectious Diseases, Oita University, Yufu, Japan; Xiamen University - Malaysia Campus: Xiamen University - Malaysia, MALAYSIA

## Abstract

**Background:**

*Helicobacter pylori* treatment failure remains a challenging problem. This study aimed to identify predictive factors for successful eradication in patients following treatment failures.

**Methods:**

This was a retrospective cohort study. This study included 1,050 dyspeptic patients diagnosed with *H*. *pylori* infection at tertiary care center in Thailand between March 2014 and October 2021. Patients’ demographic data, endoscopic findings, *H*. *pylori* culture, antimicrobial susceptibility testing (AST), treatment regimens and outcomes were analysed.

**Results:**

Of 1,050 patients with *H*. *pylori* infections, 302 (28.7%) experienced treatment failure (mean age 58.4 years; 44.7% males). AST was performed in 192. Resistance was observed for metronidazole (43.2%), levofloxacin (33.9%), clarithromycin (24%), and amoxicillin (2.1%). There was no tetracycline resistance. Multidrug-resistance (MDR) was significantly more common following treatment failure (45.5% vs. 15.7%, p<0.001). Baseline characteristics were similar between treatment successes and failures. Eradication rates after first-line and second-line regimens were 71.2% and 54.5%, respectively. Medication nonadherence [**OR 36.6 (95%CI 8.65–155.03, p<0.001)**] and MDR [**OR 4.49 (95%CI 2.29–8.81, p<0.001)**] were associated with treatment failure. Over time, resistance increased for metronidazole, levofloxacin, and clarithromycin, while eradication rates with triple therapy declined. Tailored antibiotic therapy [**OR 4.92 (95%CI 1.61–14.99, p = 0.005**)] and a regimen including 4-times-daily dosing of amoxicillin (2 grams/day) [**OR 3.05 (95%CI 1.10–8.41, p = 0.032)**] were significantly associated with treatment success after first-line failure. Eradication rates when using tailored therapy and 4-times-daily dosing of amoxicillin (2 grams/day) were 91.1% and 89.4%, respectively. Performing AST before first-line therapy resulted in the highest cure rates. AST performed after multiple treatment failures was also associated with higher eradication rates compared with the group without AST (94.4% vs. 50%,p = 0.008).

**Conclusions:**

AST either before or after treatment failure correlated with a higher proportion of successful eradication. Nonadherence and the MDR infections predicted treatment failure. Tailored therapy and 4-times-daily dosing of *amoxicillin* after treatment failure were likely to be successful.

## Introduction

*Helicobacter pylori (H*. *pylori)* is a small curved Gram-negative, rod-shaped bacterium with flagella that colonizes the gastric mucous layer, triggers a host inflammatory response, and results in chronic gastritis, peptic ulcer disease, gastric mucosa-associated lymphoid tissue (MALT) lymphoma, and gastric cancer [1]. Gastric cancer is the fourth leading cause of cancer mortality worldwide [2]. *H*. *pylori* has been designated as group I human carcinogen and eradication is recommendation to prevent gastric cancer [3, 4]. Recommended first-line treatment regimens differ depending on antimicrobial resistance patterns [5]. However, increasing resistance is the major hindrance preventing successful eradication [6].

*H*. *pylori* treatment failure or unsuccessful eradication after completing one or more course(s) of standard *H*. *pylori* treatment is increasing [7]. The most common causes of eradication failure are antibiotic resistance and poor adherence to medical therapy [7]. Increasing antibiotic resistance has been observed worldwide, especially in Southeast Asia which exhibited a significant increase in resistance to all antibiotics [8]. Thailand, located in mainland Southeast Asia, has also experienced rising antibiotic resistance. The 2015 Thai guideline recommends 14-day triple therapy, 10-day concomitant therapy, and 10-day sequential therapy as first-line treatment options, while bismuth quadruple therapy is recommended for first-line treatment in patients with penicillin allergy [9]. First-line regimens containing clarithromycin might not be effective due to higher clarithromycin resistance and the guideline needs to be revised in the near future.

As antibiotic resistance increases, more patients experience refractory *H*. *pylori* infections. After failure of second-line treatment, antimicrobial susceptibility testing is recommended to be performed [9]. However, there have been limited data about antibiotic resistance and eradication rates after failure of *H*. *pylori* treatment in Thailand. This study aimed to determine predictive factors for successful eradication in patients with *H*. treatment failures as well as to gather data on current antibiotic resistance pattern in Thailand.

## Methods

### Study design

This was a retrospective cohort study conducted at tertiary care center in Thailand. The study was conducted from February 22, 2022 to February 19, 2023. Patients’ data were retrospectively collected by chart review between March 6, 2014, and October 31, 2021. The inclusion criteria were patients older than 15 years old with *H*. *pylori* infection defined as positive rapid urease test, histopathology, or culture. Each patient underwent gastroscopy and had gastric biopsies performed. Patients’ demographic data, comorbidities, endoscopic findings, *H*. *pylori* culture and antimicrobial susceptibility testing, and treatment regimens were extracted from the medical database. Regimen composition of each first-line and second-line therapy was defined as in our previous review [10]. First-line regimen selection for each patient was decided by his physician depending on history of drug allergy. Each follow-up visit for *H*. *pylori* treatment was recorded. Patients who did not have post-treatment testing to confirm eradication were excluded from this study.

The primary aim of this study was to determine predictive factors for successful eradication in patients experiencing *H*. *pylori* treatment failure. The secondary outcomes were to determine current antibiotic resistance patterns as well as trends in eradication rates of first-line regimens in Thailand.

### Diagnosis of *H*. *pylori* infection

During upper GI endoscopy, at least four biopsies from antrum and body of stomach were performed and sent for rapid urease test and histological examination. *H*. *pylori* culture was performed in some treatment-naïve patients and patients with treatment failure. *H*. *pylori* infection was defined as a positive result to either one of these three diagnostic methods.

### *H*. *pylori* culture and antimicrobial susceptibility testing

*H*. *pylori* culture and antimicrobial susceptibility testing (AST) was previously recommended after treatment failure [11]. However, the current guideline recommends performing susceptibility tests before first-line treatment to provide higher cure rates and promote responsible antibiotic use [3]. Two antral biopsies were manually ground in broth and streaked on a Mueller Hinton II agar medium using an inoculating loop. After incubation at 37°C in a microaerophilic atmosphere for 3 to 5 days, *H*. *pylori* colonies appear on the agar plate [12]. AST was then performed to determine the minimum inhibitory concentrations (MICs) of antibiotics using the Epsilometer test (E-test). The MIC, which was the lowest concentration of antibiotics that could prevent visible bacterial growth, was determined at the intersection point between the inhibition ellipse and the scale on the E-test strip [13]. The European Committee on Antimicrobial Susceptibility Testing (EUCAST) defined resistant strains by MIC values of >0.125 mg/L for amoxicillin (AMX), >0.5 mg/L for clarithromycin (CLR), >8 mg/L for metronidazole (MTZ), >1 mg/L for levofloxacin (LVX), and >1 mg/L for tetracycline (TET) [14].

### Definitions

***H*. *pylori* treatment failure** was defined as persistent *H*. *pylori* infection after completing an eradication regimen. Persistent *H*. *pylori* infection was diagnosed by a positive rapid urease test, histopathology, or culture.

**Treatment success after first-line eradication failure** was defined as a negative urea breath test or stool antigen test after completing a treatment regimen after least one failed eradication attempt.

**Multidrug-resistant *H*. *pylori* strains (MDR)** was defined as strains which were resistant to two or more classes of antibiotics.

**Tailored therapy** was defined as *H*. *pylori* treatment by using susceptibility-guided antibiotics. Patients who did not have AST performed were considered as not using tailored therapy.

**Nonadherence** was defined as taking less than 80% of prescribed medication for *H*. *pylori* eradication (e.g., <11 days of total medication for 14-day regimen) [15].

**Standard-dose proton-pump inhibitor (PPI)** is as follows: omeprazole 20 mg, esomeprazole 20 mg, rabeprazole 20 mg, lansoprazole 30 mg, dexlansoprazole 30 mg, or pantoprazole 40 mg [7].

**High-dose PPI** is defined as double dose of a standard dose mentioned above [7].

**Potassium-competitive acid blocker (P-CAB)** in this study is vonoprazan (VPZ) 20 mg given b.i.d.

### Statistical analysis

All data were analysed by using SPSS version 22 (SPSS Inc., Chicago, IL, USA). Categorical variables were analysed by Chi-square test, or Fisher’s exact test where appropriate. Continuous variables were analysed by using Student’s t-test and reported as mean ± standard deviation (SD). Univariate analysis was performed to identify predictive factors associated with successful eradication after *H*. *pylori* treatment failure. All variables with p-values <0.05 by univariate analysis entered into multivariate analysis. Multivariate analysis was then performed to determine an adjusted p-value for each strategy in order to reach the same endpoint: treatment success after first-line eradication failure. All tests were two-sided and p values of less than 0.05 were considered as statistical significance.

### Ethics statements

The study obtained ethical approval by the Human Research Ethics Committee of Thammasat University, Thailand and was conducted according to the good clinical practice guideline, as well as the Declaration of Helsinki. All data had been fully anonymized before they were accessed. Waiver of documentation of informed consent was issued by the Ethics Committee because of no more than minimal risk to study subjects. Authors did not have access to information that could identify individual participants during or after data collection.

## Results

### Demographic data of patients with *H*. *pylori* infection

**Fig 1:**
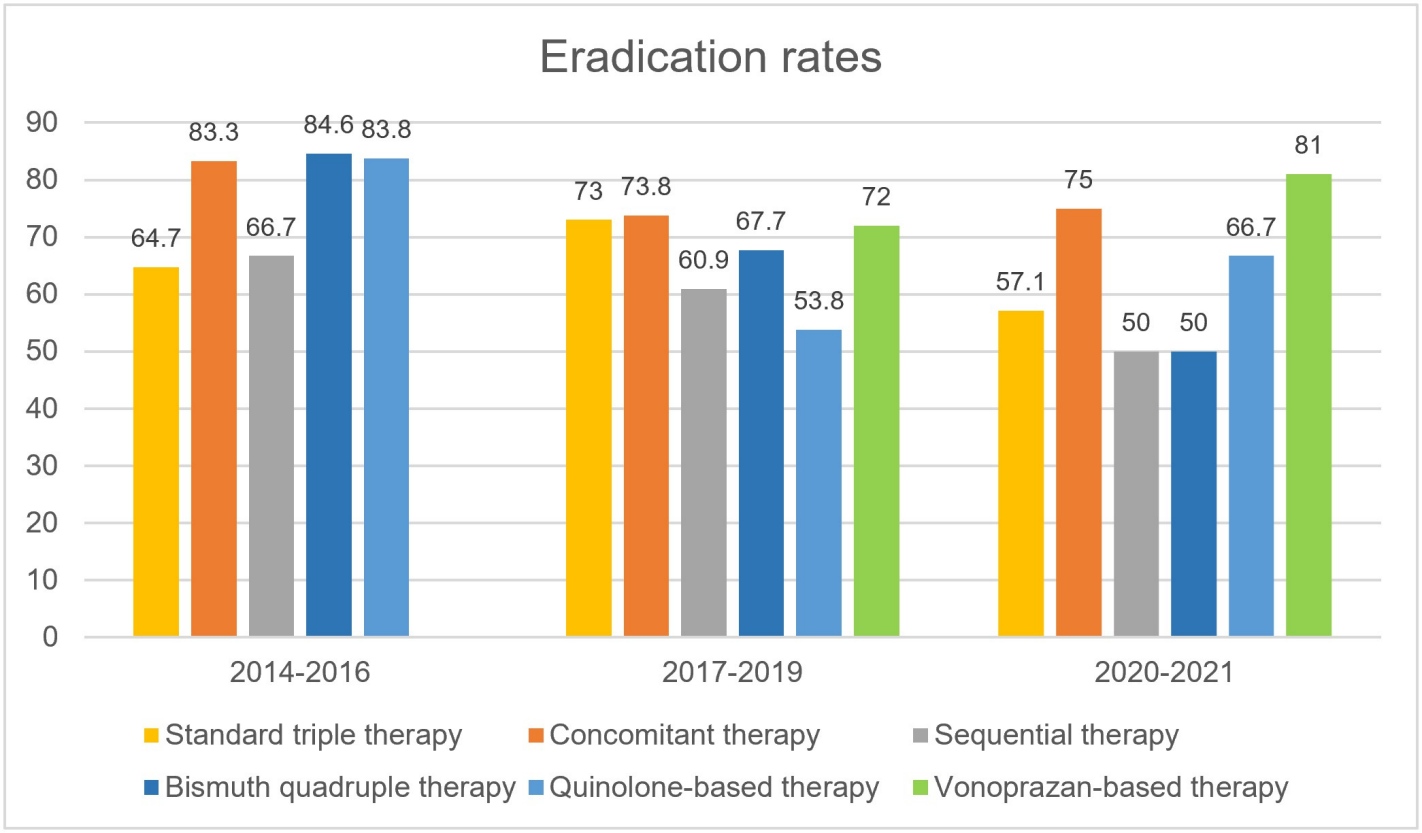
Time trends of eradication rates of each first-line regimen during 2014–2021.

During the study period, 1,840 patients underwent upper gastrointestinal endoscopy at tertiary care center in Thailand. Of 1,840 patients, there were 1,050 with *H*. *pylori* infections [471 men and 579 women; mean age 59 ± 13 (range 17–91) years] included in this study. After first-line treatment, 302 patients experienced treatment failure (28.7%); mean age = 58.4 years; 44.7% males. The baseline characteristics were comparable between treatment success and treatment failure groups (Table 1). The most common underlying diseases were hypertension (37.5%), dyslipidemia (35%), and diabetes mellitus (21%). Triple therapy (a PPI, clarithromycin, and amoxicillin for 14 days) was the most commonly prescribed first-line regimen for *H*. *pylori* eradication (39.7%), followed by bismuth quadruple therapy (14%), concomitant regimen (13.9%), levofloxacin triple therapy (12.8%), vonoprazan-containing regimens (12.8%; vonoprazan triple therapy 89/134, vonoprazan bismuth quadruple therapy 35/134, vonoprazan dual therapy 10/134), and sequential therapy (6.8%). All first-line regimens were equally distributed between treatment success and failure group except for sequential therapy which was more common in the treatment failure group. The most frequently used second-line regimens were levofloxacin triple therapy (44.9%) and bismuth quadruple therapy (27.9%). Eradication rates after first-line, second-line, and third-line regimens were 71.2% (95%CI 68.4%-74.0%), 54.5% (95%CI 48.7%-60.2%), and 44.9% (95%CI 36.1%-54.0%), respectively. Time trends demonstrated a declining eradication rate for standard triple therapy and more frequent use of vonoprazan-containing regimens since 2019 (Fig 1). Sequential therapy had an eradication rate of 50% as first-line treatment. Nonadherence to treatment and presence of MDR *H*. *pylori* strains were significantly associated with treatment failure with OR 36.6 (95%CI 8.65–155.03, p<0.001), and OR 4.49 (95%CI 2.29–8.81, p<0.001), respectively.

**Table 1:** Baseline characteristics and univariate analysis of predictors associated with treatment failure.

Characteristics	Treatment success		Treatment failure		P-value
	(N = 748)		(N = 302)		
Male	336	(44.9%)	135	(44.7%)	0.949
Age (years ± SD)	59.2 ± 12.9		58.4 ± 13.2		0.338
BMI (kg/m^2^ ± SD)	24.2 ± 4.1		24.4 ± 5.1		0.699
**Underlying diseases**					
None	225	(30.1%)	83	(27.5%)	0.403
Hypertension	281	(37.6%)	113	(37.4%)	0.964
Dyslipidemia	260	(34.8%)	108	(35.8%)	0.758
Diabetes mellitus	167	(22.3%)	53	(17.5%)	0.085
Cirrhosis and hepatitis	134	(17.9%)	45	(14.9%)	0.240
Chronic kidney disease	44	(5.9%)	22	(7.3%)	0.397
FH of gastric cancer	6	(0.8%)	3	(1.0%)	0.722
Smoking	60	(8.0%)	23	(7.6%)	0.826
Alcohol	107	(14.3%)	36	(11.9%)	0.308
**First-line treatment**					
Duration	12.1 ± 2.7		12.2 ± 2.6		0.417
Triple therapy	287	(38.4%)	130	(43.0%)	0.161
Concomitant therapy	110	(14.7%)	36	(11.9%)	0.238
Sequential therapy	44	(5.9%)	28	(9.3%)	0.049
Bismuth quadruple therapy	100	(13.4%)	47	(15.6%)	0.354
Quinolone-based therapy	103	(13.8%)	31	(10.3%)	0.123
VPZ-containing therapy	104	(13.9%)	30	(9.9%)	0.081
Nonadherence	2	(0.3%)	27	(8.9%)	<0.001
MDR strains	18/115	(15.7%)	35/77	(45.5%)	<0.001

BMI = body mass index, FH = family history, VPZ = Vonoprazan, MDR = multidrug-resistant

### Predictive factors associated with treatment success of first-line failure

After first-line eradication failure, 301 patients received a second-line treatment. Levofloxacin triple therapy and bismuth quadruple therapy were the most commonly used empiric second-line regimens as recommended by the Thai guideline [9]. Various treatment strategies were implemented to eradicate *H*. *pylori* infection. Univariate and multivariate analyses of strategies associated with treatment success after first-line eradication failure are shown in Table 2. Tailored therapy based on AST (91.1% vs. 64.9%; OR 4.92, 95%CI 1.61–14.99, p = 0.005) and 4-times-daily dosing of amoxicillin (2 grams/day) (89.4% vs. 65.7%; OR 3.05, 95%CI 1.10–8.41, p = 0.032) were significantly associated with treatment success after first-line failure. Four-times-daily dosing of amoxicillin in successful eradication regimens were demonstrated in S1 Table. Treatment duration of 14 days was associated with treatment success after first-line failure in the univariate analysis but did not reach statistical significance in the multivariate analysis. Vonoprazan was used in 24 patients (8%) for treatment after first-line failure yielding an eradication rate of 70.8%. Vonoprazan-based regimens were demonstrated in S2 Table. High-dose PPI was used in remaining patients providing a similar cure rate (69.7%). Eradication rates between regimens with or without bismuth were not different (69.2% vs. 70.2%, p = 0.853). Furazolidone-containing regimen [(PPI or VPZ) + bismuth + (AMX or TET) + furazolidone] was used for salvage therapy after multiple treatment failures providing the eradication rate of 76.9%.

**Table 2:** Eradication rates, univariate and multivariate analyses of strategies associated with treatment success after first-line eradication failure.

Strategies	Eradication rates		Univariate analysis			Multivariate analysis		
			OR	(95% CI)	p-value	OR	(95% CI)	p-value
Non-tailored therapy	159/245	(64.9%)	1					
Tailored therapy based on AST	51/56	(91.1%)	5.52	(2.12–14.34)	<0.001	4.92	(1.61–14.99)	0.005
Twice-daily dosing of AMX (2 g/day)	113/172	(65.7%)	1	-				
Four-times-daily dosing of AMX (2 g/day)	42/47	(89.4%)	4.39	(1.65–11.68)	0.003	3.05	(1.10–8.41)	0.032
High-dose PPI use	193/277	(69.7%)	1	-				
Vonoprazan use	17/24	(70.8%)	1.06	(0.42–2.64)	0.906			
No bismuth in regimen	127/181	(70.2%)	1					
Addition of bismuth	83/120	(69.2%)	0.95	(0.58–1.58)	0.853			
7- or 10-day treatment duration	41/69	(59.4%)	1					
14-day treatment duration	169/232	(72.8%)	1.83	(1.05–3.21)	0.034	1.68	(0.87–3.23)	0.121

AMX = amoxicillin, PPI = proton pump inhibitor

### Effects of antibiotic susceptibility testing on eradication rates

**Fig 2:**
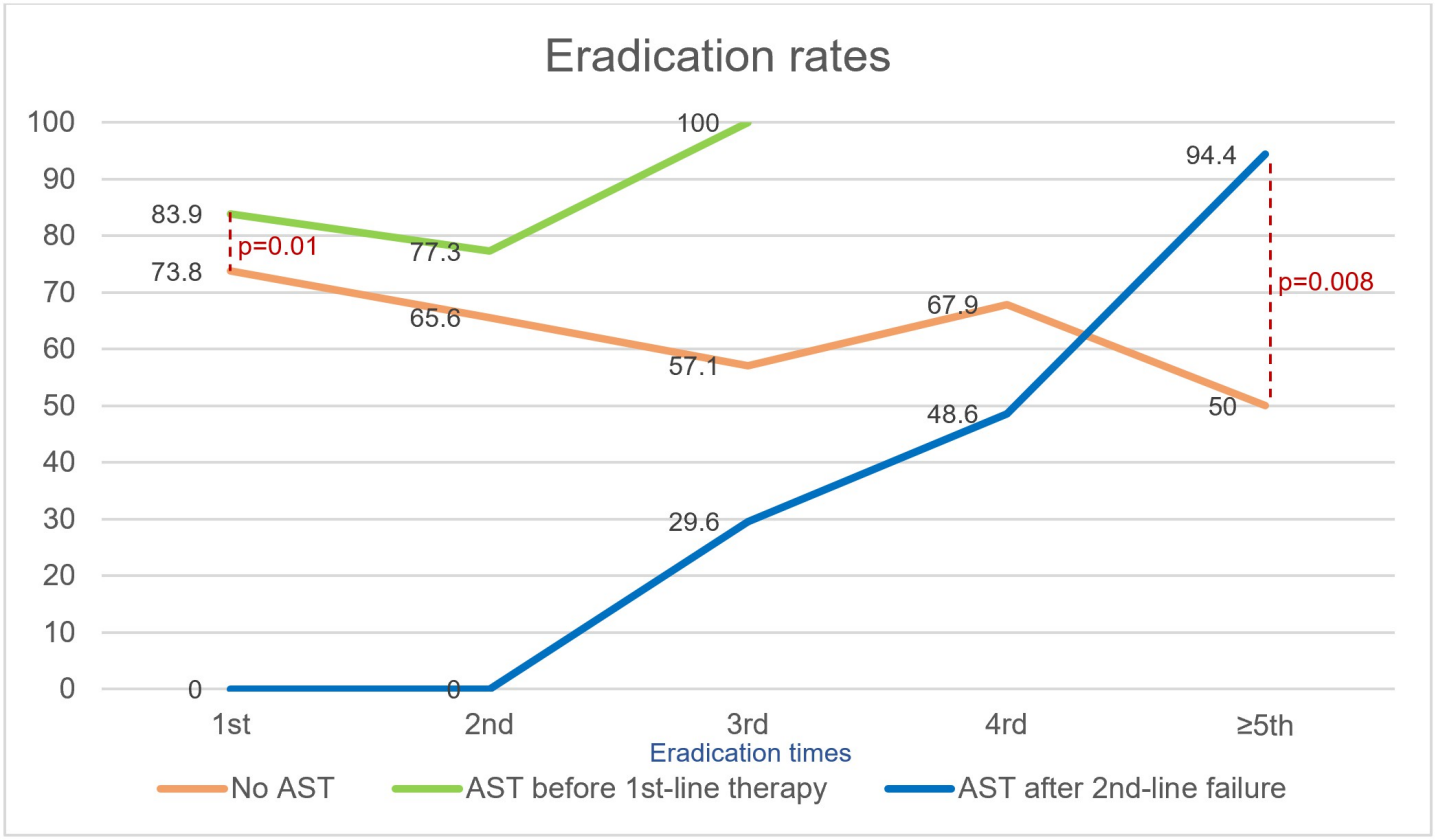
Eradication rates of 3 subgroups according to the time of performed AST. AST = Antibiotic susceptibility testing.

Patients with *H*. *pylori* infection were divided into 3 subgroups: 1) group without AST performed (n = 858); 2) group with AST performed before the first-line treatment (n = 137); 3) group with AST performed after failure of second-line therapy (n = 55). Those who had AST performed before either first therapy or second therapy had higher eradication rates (83.9% vs. 73.8%, p = 0.01) and (77.3% vs. 65.6%, p = 0.27) respectively than those without AST (Fig 2). All patients who failed second therapy who came for follow-up achieved successful eradication by the third eradication. Four patients (2.9%) in group 2 were lost to follow-up at the third eradication. The eradication rate of group 3 gradually increased after performing AST and became higher than that of group 1 after the fifth eradication (94.4% vs. 50%, p = 0.008), whereas eradication rates of group 1 decreased from 73.8% to 50% after fifth-line treatment.

### *H*. *pylori* antibiotic resistance

**Fig 3:**
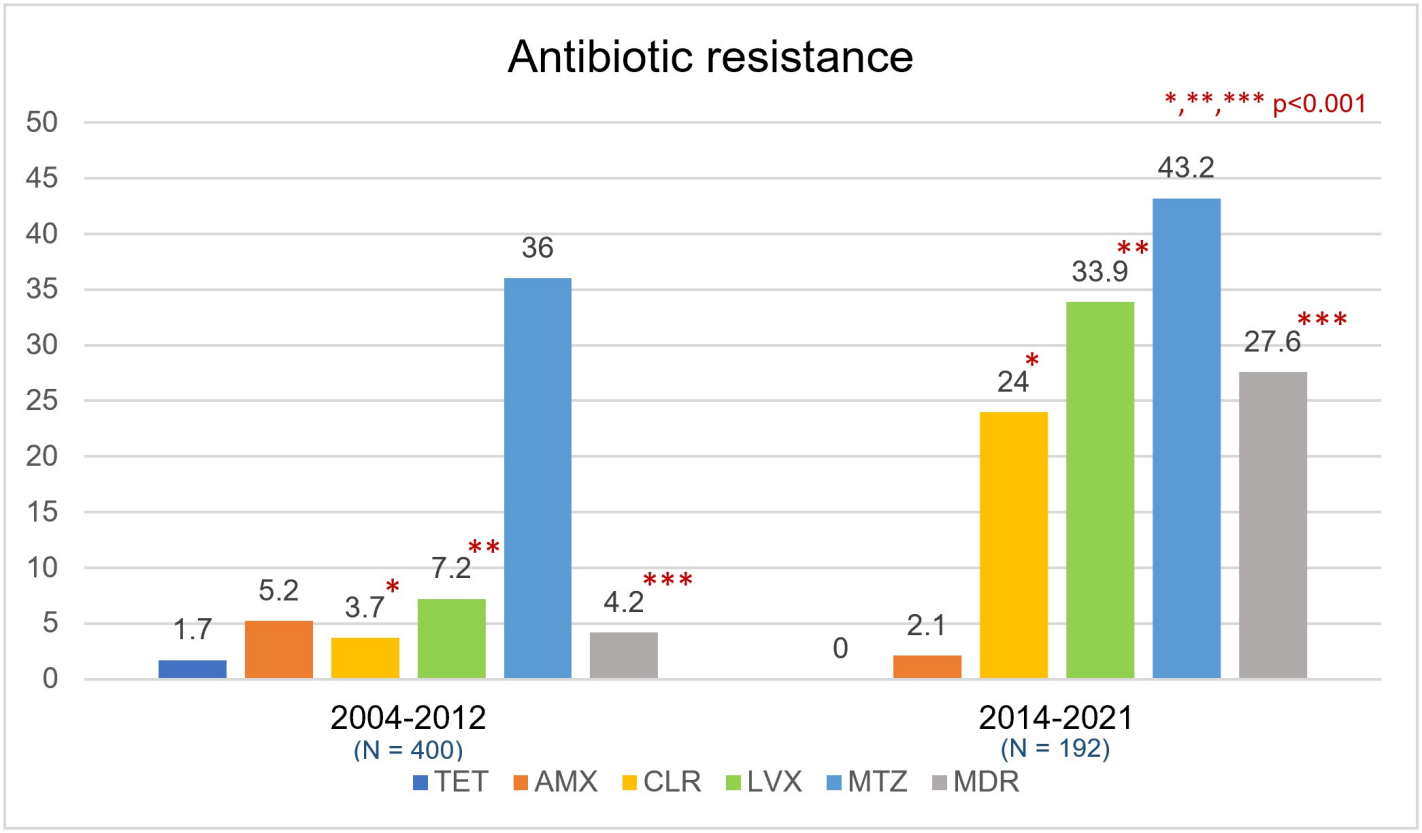
Antibiotic resistance in Thailand during 2014–2021 compared with 2004–2012. TET = Tetracycline, AMX = Amoxicillin, CLR = Clarithromycin, LVX = Levofloxacin, MTZ = Metronidazole, MDR = Multidrug-resistant strains.

AST was performed for 192 strains (Table 3). The resistance rates to metronidazole, levofloxacin, clarithromycin, and amoxicillin were 43.2%, 33.9%, 24%, and 2.1%, respectively. There was no tetracycline resistance (n = 192). In subgroup of clarithromycin-resistant strains, almost all patients receiving triple therapy, concomitant therapy, and sequential therapy had first-line eradication failure (29/31, 93.5%). Seventy strains (36.5%) had no antibiotic resistance and 53 (27.6%) were MDR strains. MDR strains were significantly more common after treatment failure (45.5% vs. 15.7%, p<0.001). An eradication rate of first-line therapy for MDR strains was only 34%. Seventeen strains (8.9% of all strains) in treatment failure group were resistant to clarithromycin, metronidazole, and levofloxacin and were eradicated by amoxicillin plus tetracycline (5/17), amoxicillin or tetracycline plus furazolidone (5/17), amoxicillin or tetracycline plus sitafloxacin (3/17), amoxicillin or tetracycline plus metronidazole (2/17), and amoxicillin plus moxifloxacin (1/17). One patient with CLR, MTZ, and LVX resistance lost to follow-up after 4^th^-line treatment. Compared with 2004–2012, CLR, LVX, and multidrug resistance substantially increased during 2014–2021 (p<0.001) (Fig 3).

**Table 3:** Antibiotic resistance pattern of *H*. *pylori* strains in patients with treatment failure.

Antibiotic resistance	Total patients		Treatment success		Treatment failure		P-value
	(N = 192)		(N = 115)		(N = 77)		
**No resistance**	**70**	**(36.5%)**	**58**	**(50.4%)**	**12**	**(15.6%)**	**<0.001**
**Antibiotic resistance**	**122**	**(63.5%)**	**57**	**(49.6%)**	**65**	**(84.4%)**	**<0.001**
Metronidazole (MTZ)	83	(43.2%)	42	(36.5%)	41	(53.2%)	0.022
Levofloxacin (LVX)	65	(33.9%)	23	(20.0%)	42	(54.5%)	<0.001
Clarithromycin (CLR)	46	(24.0%)	11	(9.6%)	35	(45.5%)	<0.001
Amoxicillin (AMX)	4	(2.1%)	1	(0.9%)	3	(3.9%)	0.304
Tetracycline (TET)	0	(0%)	0	(0%)	0	(0%)	-
**Single drug resistance**	**69**	**(35.9%)**	**39**	**(33.9%)**	**30**	**(39.0%)**	**0.475**
MTZ	38	(19.8%)	26	(22.6%)	12	(15.6%)	0.231
LVX	23	(12.0%)	9	(7.8%)	14	(18.2%)	0.030
CLR	8	(4.2%)	4	(3.5%)	4	(5.2%)	0.716
**Multidrug resistance**	**53**	**(27.6%)**	**18**	**(15.7%)**	**35**	**(45.5%)**	**<0.001**
MTZ and LVX	14	(7.3%)	10	(8.7%)	4	(5.2%)	0.360
MTZ and CLR	9	(4.7%)	4	(3.5%)	5	(6.5%)	0.488
CLR and LVX	8	(4.2%)	2	(1.7%)	6	(7.8%)	0.062
CLR, MTZ, and LVX	18	(9.4%)	1	(0.9%)	17	(22.1%)	<0.001
AMX, CLR, and MTZ	2	(1.0%)	0	(0%)	2	(2.6%)	0.160
AMX, MTZ, and LVX	1	(0.5%)	1	(0.9%)	0	(0%)	1.000
AMX, CLR, MTZ, and LVX	1	(0.5%)	0	(0%)	1	(1.3%)	1.000

## Discussion

*H*. *pylori* treatment failure has become an important global issue. Traditionally, clarithromycin resistance has been low and patients in this study mainly received clarithromycin triple therapy as first-line treatment. Most of patients with clarithromycin resistant strains receiving clarithromycin-containing regimens for first-line therapy had eradication failure. This indicates that our national guideline should be revised according to recent antibiotic resistance pattern. The mostly used second-line regimens were levofloxacin triple therapy and bismuth quadruple therapy as suggested by the Thailand consensus on *H*. *pylori* treatment [9]. Sequential therapy, which was recommended as first-line treatment in the latest guideline also had a low eradication rate (50%). Only vonoprazan-containing and concomitant regimens provided acceptable eradication rates. This is in concordance with the latest meta-analysis comparing effectiveness of first-line regimens which demonstrated high eradication rates (91.4%) when using vonoprazan triple therapy [16]. Triple therapy is currently an ineffective first-line therapy in area with high clarithromycin resistance. Despite having a well-developed guideline for *H*. *pylori* treatment in Thailand, there was heterogeneity of real-world practice and we found that most of non-gastroenterologists used triple therapy as first-line regimen. Therefore, results of this study emphasize the importance of improving medical education and implementing guideline recommendations into clinical practice. After failed second-line therapy, the eradication rate decreased to below 50% signifying the importance of achieving successful eradication with the initial therapy.

Multiple factors influencing treatment failure including host factors such as age, medication adherence, smoking, and CYP2C19 phenotype [7]. This study confirmed that medication nonadherence was significantly associated with treatment failure. Complex regimens, high pill burden, multiple dosing, and high drug costs are common adherence barriers [17]. Apart from host factors, the primary microbial factor was the presence of MDR *H*. *pylori* which was associated with a >4 times higher risk for treatment failure. For MDR strains with resistance to MTZ and other antibiotics, bismuth quadruple therapy can still be used because MTZ resistance can be overcome by using at least 1500 mg/day of MTZ for 14 days [18]. Common resistance to three classes of antibiotics (CLR, MTZ, and LVX) caused difficulty in identifying effective treatment options. The most effective regimens were a combination of AMX and TET, or AMX or TET combined with furazolidone. Rising trend of MDR strains in Thailand aligned with increasing combined CLR and MTZ resistance in many world regions [8]. Although MDR strains have been increasing rapidly in Thailand [19], we were able to achieve eradication in 92.5% of patients with MDR with susceptibility data playing a pivotal role.

After treatment failure, several strategies were used to accomplish successful *H*. *pylori* eradication. Optimized regimens provided high cure rates when guided by AST. Currently, AST is recommended to be performed prior to first-line treatment to achieve high eradication rates [3]. However, if resources are limited, AST can be performed after an initial empirical treatment failure. Our study demonstrated that tailored therapy based on AST was significantly associated with treatment success after first-line failure and should be considered in management of multiple treatment failures in Asia. Subgroup analysis revealed that the group with AST performed before first-line treatment had the best eradication rates of between 80% and 100%. Eradication failure after first-line tailored therapy might be resulted from other factors such as medication nonadherence and CYP2C19 extensive metabolizer. Moreover, endosymbiosis between *H*. *pylori* and *Candida* species might be one of mechanisms that could facilitate intracellular protection of *H*. *pylori*. [20] Eradication rates of the group with AST performed after second-line failure increased and were eventually higher than the group without AST by fifth-line treatment. On the contrary, the group without AST experienced a gradual decline of eradication rate from 73.8% to 50% after multiple eradication attempts. It can be inferred that performing AST either before first-line therapy or after multiple treatment failure would shift trends toward more successful eradications. AST must be coupled with knowledge of which therapies are highly effective locally and with high adherence to be successful. Four-times-daily dosing of amoxicillin (500 mg every 6 hours) with high dose PPI was another factor possibly contributing to treatment success after first-line failure. In this study high dose was defined as double dose rather than use of dosing and PPI proven to provide high antisecretory effect. Frequent amoxicillin dosing schemes (≥3 times daily) can also provide longer time above minimum inhibitory concentration (MIC) [21]. CYP2C19 polymorphisms also have an impact on PPI metabolism contributing to change in intragastric pH, and affect *H*. *pylori* eradication when using pH-sensitive antibiotics [22]. All patients in this study used double dose PPI or vonoprazan to achieve adequate acid suppression [7]. Importantly, there was no difference of eradication rate whether using double dose PPI or vonoprazan. The addition of bismuth could also produce synergistic effect with antibiotics and improve eradication rates [23]. However, there was no difference between group with or without bismuth. Clearly, AST alone is insufficient to reliably achieve high cure rates as it must be coupled with locally proven effective antibiotic therapies.

Rising antibiotic-resistant *H*. *pylori* has posed a worrisome threat to public health [6, 8]. The majority of strains (63.5%) in this study were resistant to at least one class of antibiotic. The key to achieving high cure rate is prescribing a therapy which provides a local eradication rate of at least 90% [24]. CLR, LVX, and MTZ resistance in this study were all higher than 15%. Compared with other countries in the Association of Southeast Asian Nations (ASEAN), *H*. *pylori* strains in Thailand demonstrated relatively higher CLR, LVX, and MTZ resistance. ASEAN countries with high CLR, LVX, and MTZ resistance include Vietnam, Philippines, Cambodia, and Thailand [25–27]. MDR strains were most often found in Cambodia (76.4%). ASEAN countries had strains with low tetracycline and amoxicillin resistance except for Vietnam [28–32]. The prevalence of *H*. *pylori* antibiotic resistance in Southeast Asian countries is shown in Table 4. In countries which had high LVX and MTZ resistance, but low CLR resistance such as Malaysia, Indonesia, and Myanmar, CLR-containing empiric regimens might still be used. In contrast, susceptibility-based therapy would be required to be able to use CLR, MTZ, and LVX in Thailand, Philippines, Cambodia, and Vietnam. Either bismuth quadruple therapy or high dose PPI plus amoxicillin can still be used provided that it has been optimized for use locally.

**Table 4:** The prevalence of *H*. *pylori* antibiotic resistance in Southeast Asian countries.

Countries, year	Number of patients	TET	AMX	CLR	LVX	MTZ	MDR	References
Vietnam, 2019	2,318	17.9	15.0	34.1	27.9	69.4	48.8	Khien et al. [25]
Philippines, 2019	42	0	0	28.6	61.9	40.5	N/A	Yumang et al. [26]
Cambodia, 2019	55	0	9.1	25.5	67.3	96.4	76.4	Tuan et al. [27]
Thailand, 2022	192	0	2.1	24.0	33.9	43.2	27.6	This study
Singapore, 2016	708	7.6	4.1	17.1	14.7	48.2	13.5	Ang et al. [28]
Laos, 2016	31	N/A	N/A	12.6	13.4	N/A	N/A	Vannarath et al. [29]
Malaysia, 2019	59	0	0	12.2	17.1	56.1	40.9	Hanafiah et al. [30]
Indonesia, 2016	77	2.6	5.2	9.1	31.2	46.7	24.7	Miftahussurur et al. [31]
Myanmar, 2022	65	0	4.6	7.7	33.8	80.0	33.8	Subsomwong et al. [32]

TET = Tetracycline, AMX = Amoxicillin, CLR = Clarithromycin, LVX = Levofloxacin, MTZ = Metronidazole, MDR = Multidrug-resistant strains, N/A = Not available

Our study has several strengths. This is a large population-based study demonstrating predictors for successful eradication in patients experiencing *H*. *pylori* treatment failure. Furthermore, this study suggests eradication strategies and raises awareness of appropriate antibiotic use by reporting the current situation of antibiotic-resistant *H*. *pylori* strains. However, our study also had some limitations. First, there was heterogeneity of real-world practice despite having a well-developed guideline for *H*. *pylori* treatment. Prescribed first-line regimens were highly varied depending on physicians’ preference. Second, this was a retrospective cohort study. Therefore, there were some missing data of culture and AST. We tried our best to perform culture and AST as much as possible and collect all positive culture results in our analysis.

In conclusion, *H*. *pylori* treatment failure has recently become a global challenging problem. Medication nonadherence and development of MDR were the primary factors predictive of treatment failure. Tailored therapy and 4-times-daily dosing of amoxicillin in treatment regimen should be considered in management of multiple treatment failures. Performing early AST after treatment failures provides higher eradication rates and should be suggested in future clinical practice, especially in the antibiotic resistance area.

## Supporting information

S1 Table Four-times-daily dosing of amoxicillin in successful eradication regimens (N = 42). (DOCX)

S2 Table Vonoprazan-containing regimens used after first-line failure. (DOCX)

S1 Checklist STROBE statement—Checklist of items that should be included in reports of observational studies. (DOCX)
