# Supplementary material for: Management of Helicobacter pylori treatment failures: A large population-based study (HP treatment failures trial)
Source: PLoS One. 2023 Nov 30;18(11):e0294403. doi: 10.1371/journal.pone.0294403 (PMC10688878; doi:10.1371/journal.pone.0294403)
Supplement: S1 Table — (DOCX) [file pone.0294403.s001.docx]

**S1 Table.** Four-times-daily dosing of amoxicillin in successful eradication regimens (N = 42)

| **Antibiotic resistance** | **Regimens with AMX (500 mg qid)** | **Number of patients** |
| --- | --- | --- |
| LVX (N = 5) | VPZ + AMX + CLR + Bismuth | 2 |
|  | PPI + AMX + MTZ + Bismuth | 1 |
|  | Triple therapy | 1 |
|  | Concomitant therapy | 1 |
| MTZ (N = 3) | PPI + AMX + FRZ + Bismuth | 2 |
|  | Triple therapy + Bismuth | 1 |
| CLR (N = 2) | PPI + AMX + MFX + Bismuth | 1 |
|  | PPI + AMX + SFX + Bismuth | 1 |
| MTZ + CLR (N = 1) | PPI + AMX + CLR + Bismuth | 1 |
| MTZ + LVX (N = 1) | PPI + AMX + TET + Bismuth | 1 |
| CLR + LVX (N = 1) | PPI + AMX + FRZ + Bismuth | 1 |
| AMX + CLR + MTZ (N = 1) | PPI + AMX + TET + Bismuth | 1 |
| CLR + MTZ + LVX (N = 8) | PPI + AMX + FRZ + Bismuth | 3 |
|  | PPI + AMX + TET + Bismuth | 2 |
|  | VPZ + AMX + SFX + Bismuth | 2 |
|  | VPZ + AMX + MFX + Bismuth | 1 |
| No resistance (N = 4) | LVX triple therapy | 2 |
|  | VPZ-AMX dual therapy | 1 |
|  | VPZ + AMX + FRZ | 1 |
| Negative culture (N = 16) | LVX triple therapy | 14 |
|  | PPI + AMX + MTZ + Bismuth | 1 |
|  | Concomitant therapy | 1 |

AMX = Amoxicillin, CLR = Clarithromycin, FRZ = Furazolidone, LVX = Levofloxacin, MFX = Moxifloxacin, MTZ = Metronidazole,

SFX = Sitafloxacin, TET = Tetracycline, VPZ = Vonoprazan
