# Supplementary material for: Management of Helicobacter pylori treatment failures: A large population-based study (HP treatment failures trial)
Source: PLoS One. 2023 Nov 30;18(11):e0294403. doi: 10.1371/journal.pone.0294403 (PMC10688878; doi:10.1371/journal.pone.0294403)
Supplement: S2 Table — (DOCX) [file pone.0294403.s002.docx]

**S2 Table.** Vonoprazan-containing regimens used after first-line failure

| **Regimens with successful eradication**  **(N = 17)** | | | **Regimens with failed eradication**  **(N = 7)** | | |
| --- | --- | --- | --- | --- | --- |
| Vonoprazan triple therapy | 4 | (23.5%) | Vonoprazan triple therapy | 3 | (42.8%) |
| Vonoprazan triple therapy plus bismuth | 3 | (17.6%) | Vonoprazan AMX dual therapy | 1 | (14.3%) |
| Vonoprazan bismuth quadruple therapy | 3 | (17.6%) | Vonoprazan bismuth quadruple therapy | 1 | (14.3%) |
| Vonoprazan + AMX + LVX | 2 | (11.8%) | Vonoprazan triple therapy plus bismuth | 1 | (14.3%) |
| Vonoprazan + AMX + MFX + Bismuth | 1 | (5.9%) | Vonoprazan + AMX + MFX | 1 | (14.3%) |
| Vonoprazan + AMX + SFX + Bismuth | 1 | (5.9%) |  |  |  |
| Vonoprazan + AMX + FRZ | 1 | (5.9%) |  |  |  |
| Vonoprazan AMX dual therapy | 1 | (5.9%) |  |  |  |
| Vonoprazan + CLR + MTZ | 1 | (5.9%) |  |  |  |
