# Supplementary material for: Management of Helicobacter pylori treatment failures: A large population-based study (HP treatment failures trial)
Source: PLoS One. 2023 Nov 30;18(11):e0294403. doi: 10.1371/journal.pone.0294403 (PMC10688878; doi:10.1371/journal.pone.0294403)
Supplement: S1 Checklist — (DOCX) [file pone.0294403.s003.docx]

STROBE Statement—checklist of items that should be included in reports of observational studies

|  | | | Item No. | | Recommendation | | Page  No. | | | Relevant text from manuscript |  |
| --- | --- | --- | --- | --- | --- | --- | --- | --- | --- | --- | --- |
| **Title and abstract** | | | 1 | | (*a*) Indicate the study’s design with a commonly used term in the title or the abstract | | 2 | | | This was a retrospective cohort study. |  |
|  |  |  |  |  | (*b*) Provide in the abstract an informative and balanced summary of what was done and what was found | | 3 | | | AST either before or after treatment failure correlated with a higher proportion with successful eradication. |  |
| Introduction | | | | | | | |  | | | |
| Background/rationale | | | 2 | | Explain the scientific background and rationale for the investigation being reported | | 4 | | | The most common causes of eradication failure are antibiotic resistance and poor adherence to medical therapy. Specific data regarding *H. pylori* treatment failure in Thailand remains unavailable. |  |
| Objectives | | | 3 | | State specific objectives, including any prespecified hypotheses | | 4 | | | This study aimed to determine predictive factors for successful eradication in patients with *H.* treatment failures as well as to gather data on current antibiotic resistance pattern in Thailand. |  |
| Methods | | | | | | | |  | | | |
| Study design | | | 4 | | Present key elements of study design early in the paper | | 5 | | | This was a retrospective cohort study. |  |
| Setting | | | 5 | | Describe the setting, locations, and relevant dates, including periods of recruitment, exposure, follow-up, and data collection | | 5 | | | This was a retrospective cohort study conducted at tertiary care center in Thailand between August 2014 and October 2021. Patients’ demographic data, comorbidities, endoscopic findings, *H. pylori* culture and antimicrobial susceptibility testing, and treatment regimens were extracted from the medical database. |  |
| Participants | | | 6 | | (*a*) *Cohort study*—Give the eligibility criteria, and the sources and methods of selection of participants. Describe methods of follow-up  *Case-control study*—Give the eligibility criteria, and the sources and methods of case ascertainment and control selection. Give the rationale for the choice of cases and controls  *Cross-sectional study*—Give the eligibility criteria, and the sources and methods of selection of participants | | 5 | | | The inclusion criteria were patients older than 15 years old with *H. pylori* infection defined as positive rapid urease test, histopathology, or culture. Each follow-up visit for *H. pylori* treatment was recorded. |  |
|  |  |  |  |  | (*b*) *Cohort study*—For matched studies, give matching criteria and number of exposed and unexposed  *Case-control study*—For matched studies, give matching criteria and the number of controls per case | | - | | | - |  |
| Variables | | | 7 | | Clearly define all outcomes, exposures, predictors, potential confounders, and effect modifiers. Give diagnostic criteria, if applicable | | 5 | | | The primary aim of this study was to determine predictive factors for successful eradication in patients experiencing *H. pylori* treatment failure. The secondary outcomes were to determine current antibiotic resistance patterns as well as trends in eradication rates of first-line regimens in Thailand. |  |
| Data sources/ measurement | | | 8* | | For each variable of interest, give sources of data and details of methods of assessment (measurement). Describe comparability of assessment methods if there is more than one group | | 6-7 | | | There were treatment success and treatment failure groups measured by number of patients. |  |
| Bias | | | 9 | | Describe any efforts to address potential sources of bias | | 5-6 | | | Retrospective study is susceptible to bias. Therefore, we had a clear definition for *H. pylori* treatment failure. |  |
| Study size | | | 10 | | Explain how the study size was arrived at | | 5 | | | We included all patients with *H. pylori* infection and excluded patients who had incomplete medical record. |  |
| Quantitative variables | | 11 | | Explain how quantitative variables were handled in the analyses. If applicable, describe which groupings were chosen and why | | 7 | | Quantitative variables were analysed by using Student’s t-test and reported as mean ± standard deviation (SD). | | | |
| Statistical methods | | 12 | | (*a*) Describe all statistical methods, including those used to control for confounding | | 7 | | Categorical variables were analysed by Chi-square test, or Fisher’s exact test. Continuous variables were analysed by using Student’s t-test. Univariate and multivariate analyses were performed to identify predictive factors associated with successful eradication after *H. pylori* treatment failure. | | | |
|  |  |  |  | (*b*) Describe any methods used to examine subgroups and interactions | | - | | - | | | |
|  |  |  |  | (*c*) Explain how missing data were addressed | | 7 | | We addressed missing data on page 11. | | | |
|  |  |  |  | (*d*) *Cohort study*—If applicable, explain how loss to follow-up was addressed  *Case-control study*—If applicable, explain how matching of cases and controls was addressed  *Cross-sectional study*—If applicable, describe analytical methods taking account of sampling strategy | | - | | - | | | |
|  |  |  |  | (*e*) Describe any sensitivity analyses | | - | | - | | | |
| Results | | | | | | | | | | | |
| Participants | | 13* | | (a) Report numbers of individuals at each stage of study—eg numbers potentially eligible, examined for eligibility, confirmed eligible, included in the study, completing follow-up, and analysed | | 1 | | 1,840 patients underwent upper gastrointestinal endoscopy at tertiary care center in Thailand and 1,050 had *H. pylori* infections. | | | |
|  |  |  |  | (b) Give reasons for non-participation at each stage | | - | | - | | | |
|  |  |  |  | (c) Consider use of a flow diagram | | - | | - | | | |
| Descriptive data | | 14* | | (a) Give characteristics of study participants (eg demographic, clinical, social) and information on exposures and potential confounders | | 7-8 | | Demographic data, medication and antibiotic resistance were demonstrated in Table 1. | | | |
|  |  |  |  | (b) Indicate number of participants with missing data for each variable of interest | | 11 | | There were 4 patients who lost to follow-up at the third eradication. | | | |
|  |  |  |  | (c) *Cohort study*—Summarise follow-up time (eg, average and total amount) | | - | | - | | | |
| Outcome data | | 15* | | *Cohort study*—Report numbers of outcome events or summary measures over time | | 8-9 | | After first-line treatment, 302 patients experienced treatment failure (28.7%), while 748 had treatment success. | | | |
|  |  |  |  | *Case-control study—*Report numbers in each exposure category, or summary measures of exposure | | - | | - | | | |
|  |  |  |  | *Cross-sectional study—*Report numbers of outcome events or summary measures | | - | | - | | | |
| Main results | | 16 | | (*a*) Give unadjusted estimates and, if applicable, confounder-adjusted estimates and their precision (eg, 95% confidence interval). Make clear which confounders were adjusted for and why they were included | | 10 | | Unadjusted estimates and confounder-adjusted estimates, and 95% confidence interval were demonstrated in table 2. | | | |
|  |  |  |  | (*b*) Report category boundaries when continuous variables were categorized | | - | | - | | | |
|  |  |  |  | (*c*) If relevant, consider translating estimates of relative risk into absolute risk for a meaningful time period | | - | | - | | | |
| Other analyses | 17 | | Report other analyses done—eg analyses of subgroups and interactions, and sensitivity analyses | | | - | | | - | | |
| Discussion | | | | | | | | | | | |
| Key results | 18 | | Summarise key results with reference to study objectives | | | 14 | | | This study confirmed that medication nonadherence was significantly associated with treatment failure. Presence of MDR *H. pylori* which was associated with a >4 times higher risk for treatment failure. | | |
| Limitations | 19 | | Discuss limitations of the study, taking into account sources of potential bias or imprecision. Discuss both direction and magnitude of any potential bias | | | 14 | | | AST must be coupled with knowledge of which therapies are highly effective locally and with high adherence to be successful. | | |
| Interpretation | 20 | | Give a cautious overall interpretation of results considering objectives, limitations, multiplicity of analyses, results from similar studies, and other relevant evidence | | | 15-16 | | | Medication nonadherence and development of MDR were the primary factors predictive of treatment failure. | | |
| Generalisability | 21 | | Discuss the generalisability (external validity) of the study results | | | 15 | | | In countries which had high LVX and MTZ resistance, but low CLR resistance such as Malaysia, Indonesia, and Myanmar, CLR-containing empiric regimens might still be used. | | |
| Other information | | |  | | | | | | | | |
| Funding | 22 | | Give the source of funding and the role of the funders for the present study and, if applicable, for the original study on which the present article is based | | | 16 | | | This study was supported by Thailand Science Research and Innovation Fundamental Fund, Bualuang ASEAN Chair Professorship at Thammasat University, and Center of Excellence in Digestive Diseases, Thammasat University, Thailand. | | |

*Give information separately for cases and controls in case-control studies and, if applicable, for exposed and unexposed groups in cohort and cross-sectional studies.

**Note:** An Explanation and Elaboration article discusses each checklist item and gives methodological background and published examples of transparent reporting. The STROBE checklist is best used in conjunction with this article (freely available on the Web sites of PLoS Medicine at http://www.plosmedicine.org/, Annals of Internal Medicine at http://www.annals.org/, and Epidemiology at http://www.epidem.com/). Information on the STROBE Initiative is available at www.strobe-statement.org.
